# Supplementary material for: A set of multi-entry identification keys to African frugivorous flies (Diptera, Tephritidae)
Source: Zookeys. 2014 Jul 24;(428):97–108. doi: 10.3897/zookeys.428.7366 (PMC4143993; doi:10.3897/zookeys.428.7366)
Supplement: Supplementary material 6 — Key to Ceratitis [file zookeys-428-097-s006.zip › SF6_ZooKeys_key to Ceratitis/key/SF6_ZooKeys_key to Ceratitis/Media/Html/Ceratitis rosa .htm]

Ceratitis (Pterandrus) rosa Karsch


***Ceratitis*** **(*Pterandrus*) *rosa* Karsch**

*Ceratitis* *rosa* Karsch, 1887: 1.

Body
length: 4.96 (4.25-5.30) mm; wing length: 5.34 (4.50-5.75) mm.

Male

Head: Antenna yellow. First flagellomere 2-3 times as long
as pedicel. Arista with short to moderately long rays; ventral rays shorter and
sparser than dorsal rays, especially basally. Frons yellow; with short
scattered setulae distinctly darker than frons. Frontal setae well developed.
Face yellowish white. Genal seta and setulae dark, well developed.

Thorax:
Postpronotal lobe yellowish white, without spot, although sometimes darker
yellow around postpronotal seta. Scutal pattern: ground color grayish-brown
with orange tinge; with streaks and darker markings but without distinct spots
except prescutellar white markings separate, usually with paler area in
between. Scapular setae dark. Scutellum yellowish white, basally usually with
two separate dark spots, sometimes less distinct; apically with three separate
black spots, extending to basal 0.33. Anepisternum on ventral half darker
yellowish brown; setulae pale.

Legs:
Yellow except where otherwise noted; setation typical for subgenus, mainly
pale. Foreleg: femur without bushy feathering posteriorly, only dispersed rows
of long black setulae posterodorsally, posteroventrally shorter and
pale; ventral setae black. Midleg: femur with few dispersed pale setulae
ventrally; tibia moderately broadened; anteriorly black with conspicuous
silvery shine when viewed from certain angle on distal 0.66 to 0.75 (black
color sometimes inconspicuous in teneral specimens but silvery shine is always
present) with black feathering dorsally along distal 0.75 and ventrally along
distal 0.66, ocassionaly to distal 0.75. Hindleg: femur at apical 0.25 with
longer setulae dorsally and ventrally.

Wing:
banding yellowish brown. Interruption between marginal and discal bands
near vein R_1_ clear and complete; cubital band free; medial band
absent; crossvein R-M opposite middle of discal cell. Apex of vein R_1_ distal
to level of crossvein R-M. Crossvein DM-Cu oblique anterobasally.

Abdomen:
Mostly yellow. Tergites 2 and 4 with pale gray band on posterior half, anterior
margin sometimes with narrowly brownish colored, especially laterally. Tergite
3 with posterior half patchily brownish colored, anterior half yellowish brown,
both parts not clearly demarcated; sometimes more complete brown. Tergite 5
with basal half brownish, sometimes divided medially into two spots. Male
epandrium in lateral view with posterior lobe of lateral surstylus short and
straight, anterior lobe well pronounced.

Female

As
male except for the following characters: First flagellomere yellowish orange.
Crossvein DM-Cu oblique posterobasally. Anepisternum on ventral part rarely
with darker setulae. Legs without feathering; forefemur posteroventrally with
pale pilosity, at least basally. Oviscape shorter than preabdomen. Aculeus at
most six times longer than wide; tip with distinct apical indentation and
lateral margin slightly sinuous (specimens from Luabo, Mozambique seem to have
an aculeus tip which is without any indentation but otherwise completely fit
the description of *rosa*. They are tentatively placed here).

(Description
after De Meyer & Freidberg, 2006)
